# Quality of life for post gastric sleeve surgery patients: a cross sectional study

**DOI:** 10.1186/s12893-026-03630-8

**Published:** 2026-03-23

**Authors:** Karima Adel Mohamed, Nadia Hamed Farahat, Walaa Abdelkader Mohamed

**Affiliations:** Family and Community Health Nursing, Faculty of Nursing, Ain Shams University, Cairo, Egypt

**Keywords:** Bariatric surgery, Obesity, Postoperative practices, Quality of life, Sleeve gastrectomy

## Abstract

**Background:**

The prevalence of the global obesity epidemic continues to rise, and sleeve gastrectomy is widely recognized as one of the most successful treatments for morbid obesity. It results in considerable and long-lasting weight loss, remission of obesity-related health conditions, and long-term improvement in quality of life (QoL). This study aimed to primarily examine the correlation between patients’ reported postoperative practices and their quality of life following sleeve gastrectomy.

**Methods:**

A cross-sectional descriptive correlational study was performed at the bariatric surgery outpatient clinic at El-Demerdash Hospital in Cairo Governorate, Egypt. A purposive sample of 128 patients who met the required criteria was included. Data were collected via two instruments: a structured interviewing questionnaire and the WHOQOL scale. Statistical analysis was performed using SPSS (Version 22). The quantitative data are expressed as the mean± standard deviation. The Pearson’s correlation coefficient (r) was used to examine correlations between continuous variables. Statistical significance was considered at *p* < 0.05, and high statistical significance at *p* < 0.001.

**Results:**

Among participants, 57.9% had satisfactory knowledge regarding obesity, and 64% had satisfactory knowledge about sleeve gastrectomy, the majority of participants demonstrated good adherence to postoperative practices across several domains. Constipation appeared to be the most common long-term complaint. Overall, 63.3% of patients demonstrated a good level of QoL. A statistically significant positive correlation was found between total postoperative practice scores and total QoL scores (*p* < 0.001).

**Conclusion:**

Most patients who underwent sleeve gastrectomy had satisfactory knowledge and adherence to postoperative practices which positively affected their QoL, whereas only a minority experienced long-term complications. Structured educational programs are recommended for patients undergoing sleeve gastrectomy to correct misconceptions, particularly regarding dietary restrictions, promote adherence to postoperative guidelines, and consequently improve their overall quality of life.

## Background

Obesity is a major global public health challenge and a leading contributor to preventable morbidity and mortality. It is currently understood as a multifactorial chronic disease influenced by biological, behavioral, environmental, and social determinants [1–2]. In Egypt, recent national survey “100 million health” indicate that obesity represents a significant public health burden, affecting approximately 40% of adults, with a higher prevalence among women, highlighting the growing burden on the healthcare system [3].

Bariatric surgery has been shown to provide superior long-term outcomes in weight loss and management of obesity related clinical parameters compared with non-surgical treatment options [4].

Among available bariatric procedures, sleeve gastrectomy remained the most commonly performed procedure worldwide, reflecting its widespread adoption and favorable outcomes [5]. In recent years, Egypt has witnessed a marked increase in the number of bariatric procedures performed across different centers. The overall volume of bariatric interventions expanded between 2021 and 2023, with a marked rise in sleeve gastrectomy, reflecting the growing national burden of obesity [6]. Despite the growing volume of bariatric surgeries in Egypt, postoperative follow-up has revealed considerable variability in patient-reported outcomes.

Quality of life is increasingly recognized as a key patient reported outcome following bariatric surgery [7]. Growing evidence suggests that weight reduction alone does not fully reflect postoperative success. Despite comparable surgical outcomes, patients report substantial variability in postoperative quality of life, suggesting that factors beyond the surgical procedure itself influence recovery and well-being. Among these factors, adherence to recommended postoperative practices including dietary modification, physical activity, supplementation, and regular follow-up may play a crucial role. However, evidence examining the relationship between postoperative practices and quality of life remains limited, particularly in the Egyptian context [8]. Therefore, this study aimed to explore the correlation between patients’ reported postoperative practices and their quality of life following sleeve gastrectomy.

### Aims of the study

This study primarily aimed to examine the correlation between patients’ reported postoperative practices and their quality of life following sleeve gastrectomy. The secondary objectives were to:

1. Assess patients’ knowledge regarding obesity and sleeve gastrectomy.
2. Assess reported postoperative practices related to dietary habits, physical activity, and follow-up care.
3. Identify common immediate and long-term postoperative health problems following sleeve gastrectomy.

### Research question

Is there a correlation between patients’ reported postoperative practices and their quality of life after sleeve gastrectomy?

## Methods

### Study design

A cross-sectional descriptive correlational study design was employed to conduct this study. The study is reported in accordance with the STROBE guidelines for cross-sectional studies.

### Study setting

The study was performed at the bariatric surgery outpatient clinic at El-Demerdash Hospital, Ain Shams University, Cairo, Egypt, between November 2024 and March 2025.

### Participants and sample size

A purposive sample of 128 patients was selected from an accessible population of 192 postoperative sleeve gastrectomy patients attending the bariatric surgery outpatient clinic in the previous year. The target sample size of 128 was calculated using the Thompson [9] formula, assuming a probability of 0.5 for the characteristic of interest, which is a conservative estimate to maximize sample size and ensure adequate power.

$$\mathrm{Sample}\:\mathrm{equation}=\frac{N\:\times\:p(1-p)}{[N-1\times\left(d2\:\div\:Z2\right)]+P\left(1-P\right)}$$

where:

P (probability): (0.5) The assumed probability of the characteristic of interest in the population.

1 – P (Complement Probability): (0.5) The probability of not having the characteristic.

D (rate of error): (0.05) The maximum acceptable difference between the sample result and the true population value.

Z (Z-score): (1.96) The standard score corresponds to a 95% confidence level (significance level = 0.05).

N (Population Size): (192) The total number of individuals in the population.

Based on these parameters, the calculated sample size was: 128 patients.

Inclusion criteria: patients aged ≥ 18 years, who had undergone sleeve gastrectomy between 3 and 12 months, of either sex, and willing to participate.

Exclusion criteria: patients unable to complete the questionnaire or who refused participation.

Sample selection: From the accessible population of postoperative patients attending the outpatient clinic during the data collection period, patients were approached consecutively and screened for eligibility based on the predefined inclusion and exclusion criteria. Eligible patients who agreed to participate were enrolled until the target sample size of 128 was reached. This purposive, consecutive sampling approach was used to reduce potential selection bias.

### Data sources/ measurement

#### Two tools used for data collection

##### Tool I: structured interviewing questionnaire

This tool is composed of four parts: two parts were developed by the researcher after the researcher reviewed the related recent scientific literature [10], while the knowledge and practice sections were adapted from El-Sayed et al. [11] and written in simple Arabic language. The tool consists of four parts as follows:

Part I: To assess the sociodemographic characteristics of the studied patients, was developed by the researcher, it composed of six closed ended questions: age, sex, education level, marital status, job, and place of residence. Medical history reported by patients (past and present health history).

Part II: To assess immediate and long term health problems after surgery,2 sections were developed by the researcher, the first section included 9 questions to assess immediate health problems after surgery (sever bleeding, wound infection and complications of anesthesia). The second section assesses long term health problems after surgery and consists of 20 questions, such as obstruction of stomach, hernia, esophageal reflux and malnutrition.

Part III: To assess patients’ knowledge regarding obesity and sleeve gastrectomy this part was adapted from [11] and consisted of 16 closed ended questions as: meaning of obesity & Body Mass Index (BMI), causative factors of obesity, symptoms of obesity, morbid obesity, BMI for morbid obesity, complications of obesity, methods of treatment of obesity, meaning of surgery, aim of surgery, indications of surgery, benefits of surgery, criteria for surgery, complications of surgery, and meaning and signs of dumbing syndrome). The scoring method was modified by the researcher. Each correct answer was given a score of 1, and incorrect answers scored 0, with a total possible score of 16. Scores were converted into percentages; ≥50%(8– 16 degrees) indicated satisfactory knowledge and < 50%(< 8 degrees) indicated unsatisfactory knowledge.

Part IV: To assess patients’ reported practices after sleeve gastrectomy, it was adapted from [11] was composed of 8 closed ended questions : number of meals per day, type of food, precautions during meals, vitamins taken, beverages avoided, time taken to eat meals, weight tracking, and exercise).Six sections consisted of 51 items divided into parts as follows: diet” 18 items”, physical activity " 10 items”, sleep and rest " 5 items”, smoking " 6 items”, follow up " 5 items”, and medication adherence " 7 items”. Responses were scored as 1 = never, 2 = sometimes, 3 = always. Total scores were converted into percentages and categorized as follows: Scores < 50% were considered poor practice, 50%-75% were considered average practice, and > 75% were considered good practice.

##### Tool II: quality of life questionnaire (adopted from the WHOQOL) to assess patients’ quality of life after surgery

This instrument, which was adopted from the World Health Organization’s quality of life (WHOQoL) translated version [12] consists of 60 items and uses three categories of Likert scales as always, sometimes, and never. Seven domains were Covered: physical, psychological, social, environmental, daily activities, mental, and spiritual wellbeing. The scoring system of the quality of life scale score was calculated as (3) scores for always, (2) scores for some times and (1) scores for never. The total quality of life score was categorized as follows: >60%: good QoL ,30–60%: average QoL and < 30%: poor QoL.

#### Validity and reliability of the instrument

To ensure that the instruments measured the intended concepts, content validity procedures were followed, five specialized experts from the Family and Community Health Nursing Department affiliated with Ain Shams University, reviewed all the items to evaluate their clarity and relevance. The item-level content validity index (I-CVI) and the scale-level content validity index (S-CVI) were calculated, and the results yielding satisfactory values, with a knowledge scale score of 0.824, a practice scale score of 0.916, and the quality of life scale scoring 0.871., indicating good content validity of the instrument. Furthermore, internal consistency reliability was assessed using Cronbach’s alpha coefficient, with the knowledge scale scoring 0.870, the practice scale scoring 0.891, and the quality of life scale scoring 0.821, indicating good consistency among items and the ability of the instruments to produce reliable results. It should be noted that content validity and internal consistency represent only partial validation of the instruments, which is appropriate for the exploratory, cross-sectional design of this study.

### Ethical considerations

The study was conducted in accordance with the principles of the Declaration of Helsinki. Ethical approval was obtained from the Research Ethics Committee, Faculty of Nursing, Ain Shams University, Cairo, Egypt and the Institutional Review Board (IRB) (Code No. 2507768), in accordance with the committee’s ethical standards. A formal letter describing the study’s title and objectives was issued by the Dean of the Faculty of Nursing, Ain Shams University, and delivered to the Chief Executive Officer of Ain Shams University Hospital to request approval for data collection at the selected site. In addition, before participation, the purpose and procedures of the study were clearly explained to all participants. Written informed consent was obtained from each participant in accordance with institutional policy. All collected data and questionnaires were coded to maintain anonymity and ensure confidentiality. Participation was completely voluntary, and participants were informed that they had the right to withdraw at any stage without any consequences. The study involved no physical, psychological, social, or financial risk to participants.

### Data collection phases

Following ethical approval from the Scientific Research Ethical Committee at the Faculty of Nursing, Ain Shams University, and official permission from the relevant authorities, a pilot study was conducted with 13 participants (10% of the intended sample). The pilot study aimed to identify potential issues, refine the clarity and flow of the data collection tools, and estimate the average completion time (approximately 20–25 min). Participants in the pilot study were excluded from the main study to prevent bias in the final analysis. The main data collection was carried out over a five-month period, from November 2024 to March 2025. Eligible patients were approached during their routine postoperative follow-up visits at the outpatient bariatric clinic. All 128 invited patients agreed to participate, resulting in a 100% response rate. Interviews were conducted in a quiet and private area adjacent to the clinic’s waiting room to ensure confidentiality and patient comfort. The researcher personally conducted all interviews to maintain consistency, accuracy, and reliability of the collected data, while ensuring clear communication and accommodating patients from diverse socioeconomic backgrounds.

### Bias

Several steps were taken to minimize potential sources of bias. Selection bias was reduced by applying consecutive purposive sampling among eligible patients attending the outpatient clinic during the data collection period. Response bias was minimized, as all invited eligible patients agreed to participate (100% response rate). To minimize recall bias related to study variables (knowledge, postoperative practices, and quality of life), data were collected through structured interviews conducted in a private setting. Clarification of questions was required for a small subset of participants (*n* = 9) due to literacy difficulties. In these cases, questions were read verbatim using standardized neutral wording to minimize interviewer influence.

### Statistical analysis

Collected data were coded and analyzed using the statistical package for social sciences, version 22.0 (SPSS Inc., Chicago, Illinois, USA). The quantitative data are expressed as mean± standard deviation (SD). The Chi-square (χ²) test was applied to compare proportions of qualitative variables. The Pearson correlation coefficient (r) was used to construct the correlation matrix. Statistical significance was set at a P-value < 0.05, while a P-value < 0.001 was considered as highly significant. Multiple linear regression was used to examine the relationships between the dependent variable and multiple independent variables.

## Results

### The study’s patient frequency distribution regarding sociodemographic traits (*n* = 128)

Table 1 illustrates that participants had a mean age of 41.33 ± 4.97 years, with the majority in the 40–50 year age group. Most participants were female and had attained higher education. Nearly half were married and slightly more than half were employed, and a slightly higher proportion lived in urban area.

**Table 1:** Distribution of the sociodemographic traits of the studied patients. *N* = 128

Demographic characteristics of patients	*N*	%
Age (in years)		
From 18 > 30 years	20	15.6
From 30 > 40 years	45	35.2
From 40 > 50 years	53	**41.4**
50 years and over	10	7.8
Mean ± SD	**4** **1.33 ± 4.97**	
Sex		
Male	54	42.2
Female	74	**5** **7.** **8**
Educational level:		
No read or write	9	7.0
Reads and writes	11	8.6
Elementary education	6	4.7
Secondary education	33	25.8
High education	69	**53.9**
Marital status:		
Single	43	33.6
Married	60	**46.9**
Divorced	5	3.9
Widow	20	15.6
Occupation		
Employee	70	**54.7**
Not working	20	15.6
Craftsman	38	29.7
Place of residence:		
Urban	73	**57.0**
Rural	55	43.0

### Past and present history of the studied patients

Table 2 presents the pre- and post-operative medical history of participants. Before surgery, most participants weighed over 100 kg, with a mean weight of 112.33 ± 6.30 kg, and a majority had at least one chronic condition, mainly heart disease and diabetes. Most participants had previously attempted weight-loss strategies, often resulting in weight regain. Regarding the current status, nearly half underwent sleeve gastrectomy 6–10 months prior to data collection, with the majority achieving weight loss below 40 kg and a postoperative BMI mostly in the 40–50 kg/m² range. About one third continued to experience pre-existing health problems.

**Table 2:** Distribution of patients’ past and current medical history (*n* = 128)

Past medical history	*N*	%
Weight before having surgery		
Less 100 kg	57	44.5
Above 100 kg	71	55.5
Mean ± SD	**112.33 ± 6.30**	
Patients suffering from chronic disease	90	70.3
*If yes, The chronic disease (*n* = 90)		
Heart diseases	30	**33.3**
High levels of cholesterol in the body	9	10.0
Arthritis	10	11.1
Diabetes	23	**25.6**
Thyroid disorder	2	2.2
Chronic kidney disease	1	1.1
Hypertension	10	11.1
Stroke	0	0.0
COPD	5	5.6
Obesity onset		
Since childhood	26	20.3
Since adolescence	39	**30.5**
Since birth	20	15.6
After marriage	33	25.8
During pregnancy	10	7.8
Patients have been exposed to diseases resulting from obesity	69	53.9
*If yes, what are these problems (*n* = 69)		
Diabetes	22	31.9
Hypertension	8	11.6
High levels of cholesterol	9	13.0
Pain of joint	30	**43.5**
Follow a weight management method	90	70.3
*If yes, method to lose weight(*n* = 90)		
Diet	30	33.3
Exercise	20	22.2
Medications	33	**36.7**
Medicinal herbs	7	7.8
Diet and exercise	23	25.6
The result of these method (*n* = 90)		
Weight loss and then gain	80	**88.9**
Weight Stability	10	**11.1**
Current medical history	**N**	**%**
Sleeve gastrectomy done		
3 > 6months	28	21.9
6 > 10months	60	**46.9**
10 > 12 months	40	31.2
Weight did lose after surgery		
Less than 20 kg	55	**43.**0
20–40 kg	43	**33.6**
> 40 kg	30	**23.4**
Body mass index (BMI)		
Less than 30 kg2.	2	1.6
30 kg2 < 35 kg2.	40	31.3
35 kg2 < 40 kg2.	3	2.3
40 kg2 < 50 kg2.	83	64.8
50 kg2 or more	0	0.0
Mean ± SD	**40.63 ± 5.38**	
*The reason behind the decision to perform surgery		
Inability to practice daily activities.	35	27.4
Obesity-related diseases.	40	31.2
Appearing appropriately.	53	41.4
All of the above.	50	39.0
Patients still suffer from health problems that were present before surgery	40	31.2

*N* Number of patients

*N.B. Items not mutually exclusive

### Distribution of studied patients regarding health problems related to sleeve gastrectomy (*N* = 128)

Table 3, presents the immediate and long term postoperative health problems. Only a small proportion of participants experienced immediate complications, such as severe stomach pain, wound infection, or stomach leakage. Long-term issues were more common, with constipation and changes in stool shape being the most frequently reported, followed by malnutrition, vomiting, and iron deficiency. Overall, most participants did not experience serious postoperative complications.

**Table 3:** Distribution of studied patients regarding health problems related to sleeve gastrectomy. *N* = 128

Health problems immediately after operation	YES	
	*N*	%
Severe bleeding after the operation	0	0.0
Infection and inflammation of the wound site	2	1.6
Blood clots	0	0.0
Lung or breathing problems	1	0.8
Stomach leakage	2	1.6
Stomach inflammation	0	0.0
Severe pain or burning in the stomach	10	**7.8**
Open wound	0	0.0
Long-term health problems		
Gastrointestinal obstruction	0	0.0
Hernia cases	0	0.0
Esophageal reflux	2	1.6
Hypoglycemia	0	0.0
Malnutrition	12	9.4
Malabsorption	7	5.5
Vomiting	10	7.8
Gallstones	5	3.9
Indigestion	0	0.0
Failure to lose weight	4	3.1
Gain new weight	0	0.0
Hair loss	5	3.9
General weakness	3	2.3
Iron deficiency (anemia)	11	8.6
Low calcium level	0	0.0
Change in the shape of the stool	30	**23.4**
Constipation	55	**43.0**
Diarrhea	2	1.6

### Distribution of sleeve gastrectomy patients’ regarding total knowledge. (*n* = 128)

**Fig. 1:**
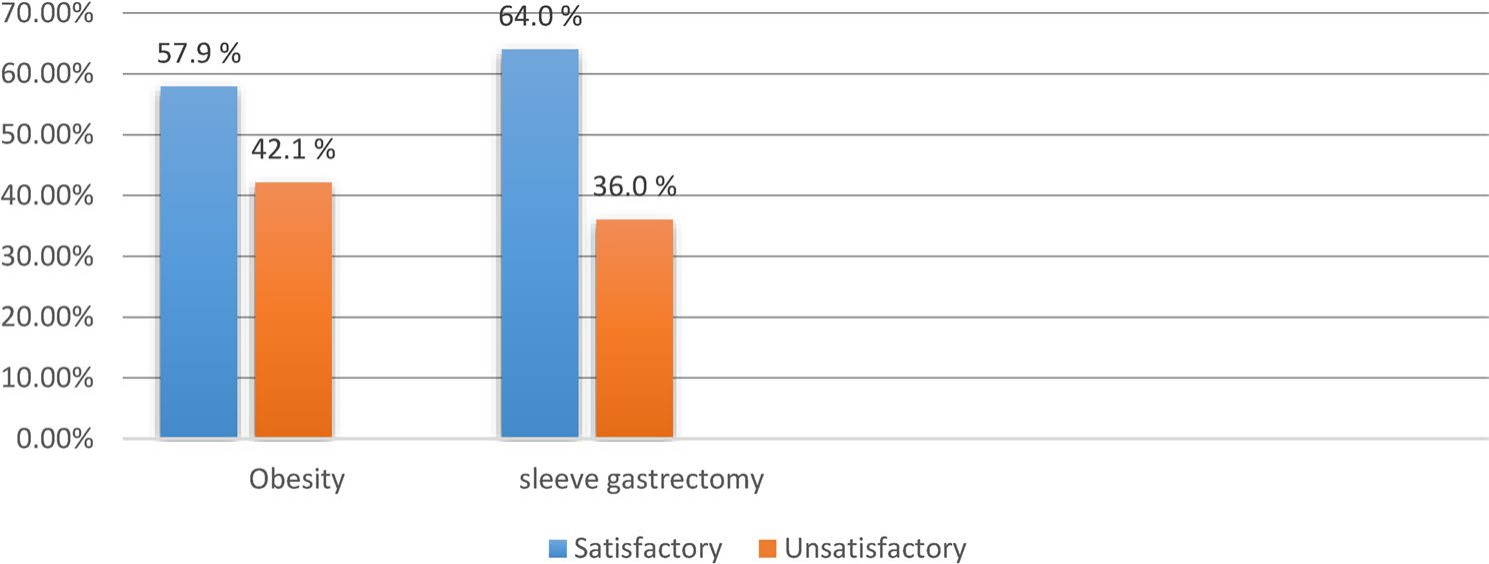
Distribution of sleeve gastrectomy patients’ regarding total knowledge. (n=128)

Figure 1 shows that a higher proportion of participants had satisfactory knowledge about sleeve gastrectomy compared to obesity.

### Distribution of sleeve gastrectomy patient’s regarding total domains of reported practices after surgery

Table 4 summarizes participants’ postoperative adherence across multiple domains. Most participants reported good practice with diet, physical activity, sleep and rest, periodic follow-up, and medication adherence. Smoking cessation was notably high among smokers. Overall, adherence to recommended postoperative practices was generally satisfactory.

**Table 4:** Distribution of sleeve gastrectomy patients regarding total domains of practice after surgery. *N* = 128

Domains	Good		average		Poor	
	*N*	%	*N*	%	*N*	%
Diet	80	62.5	28	21.9	20	15.6
Physical activities (Exercise)	90	70.3	25	19.5	13	10.2
Sleep and rest	89	69.5	29	22.7	10	7.8
Smoking	45	90.0	2	4.0	3	6.0
Periodic follow-up	90	70.3	18	14.1	20	15.6
Adherence to medication	81	63.3	23	18.0	24	18.7

### Distribution of patients’ total quality of life after surgery. *N* = 128

Table 5 presents participants’ quality of life across seven domains. Most participants reported good QOL in the social, psychological, and physical domains, while daily activities and mental domains showed slightly lower levels. Overall, the findings indicate generally favorable postoperative quality of life among the study cohort.

**Table 5:** Distribution of patients’’ total quality of life after sleeve gastrectomy. *N* = 128

Domains	Good		Average		Poor	
	*N*	%	*N*	%	*N*	%
Physical domain	80	62.5	28	21.9	20	15.6
Psychological domain	83	64.9	25	19.5	20	15.6
Social domain	86	67.2	21	16.4	21	16.4
Environmental domain	85	66.4	29	22.7	14	10.9
Daily activities domain	73	57.0	34	26.6	21	16.4
Mental domain	75	58.6	34	26.6	19	14.8
Spiritual domain	80	62.5	25	19.5	23	18.0

**Fig. 2:**
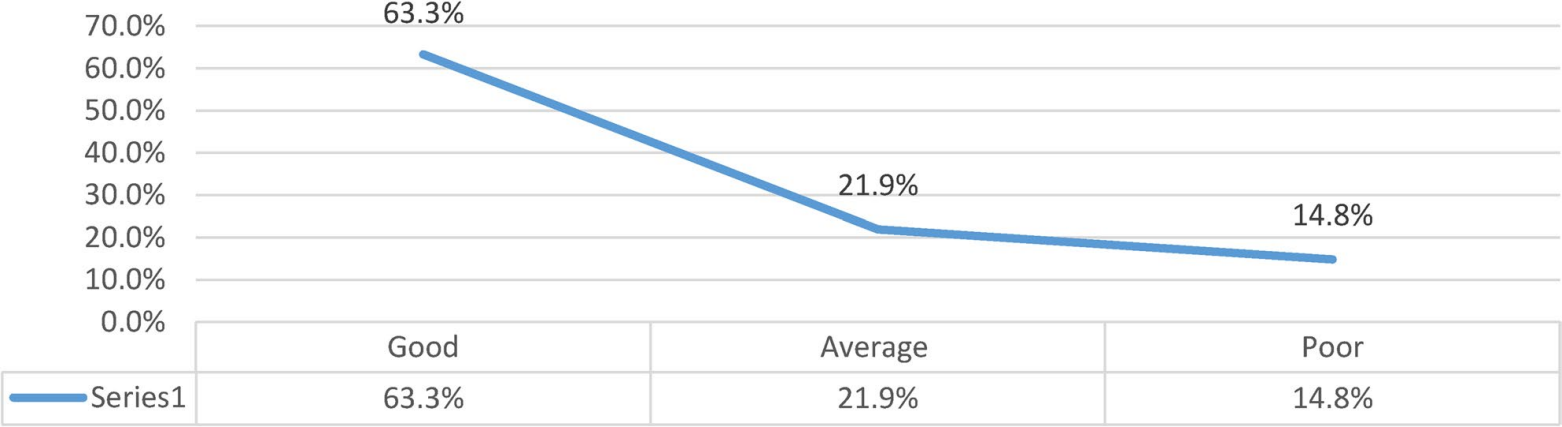
Distribution of patients’ total quality of life after surgery. N =128

Figure 2 show the majority of participants demonstrated a good level of quality of life, while smaller proportions reported average and poor levels.

### Correlation between the studied patients’ total reported practices and total quality of life

Table 6 shows a strong, statistically significant positive correlation between total reported postoperative practices scores and overall quality of life (*p* < 0.001). This suggests that greater adherence to recommended postoperative practices is associated with higher quality of life among participants.

**Table 6:** Correlation between patients’ total reported practices and total quality of life. *N* = 128.

Variable	Total quality of life	
Total reported practice	r	0.441
	P	0.000** (* =*P*<0.05, statistically significant. ** = *p*<0.001,highly significant).

### Multiple regression models for predicting patients’ baseline characteristics associated with quality of life after sleeve gastrectomy

Table 7 presents the multiple linear regression analysis identifying predictors of total quality of life among post sleeve gastrectomy patients. The overall model was statistically significant (F = 7.217, *p* < 0.001) and explained 65.1% of the variance in QoL scores (R² = 0.651). Sex, occupation, BMI, patients’ knowledge, and reported postoperative practices were significant predictors of QoL (*p* < 0.05), whereas age, educational level, marital status, and time since surgery were not significantly associated with QoL (*p* > 0.05).

**Table 7:** Best fitting multiple regression models for predictors of patients’ baseline characteristics in quality of life sleeve gastrectomy patients

Model	Unstandardized Coefficients		Standardized Coefficients	t	Sig.
	β	Std. Error	Beta		
(Constant)	4.276	1.213	0.000	9.153	< 0.001**
Age (in years)	0.122	0.132	0.142	1.821	0.215
Sex	1.026	0.391	0.194	2.493	0.016*
Educational level	0.161	0.131	0.184	0.727	0.115
Marital status	0.138	0.113	0.158	1.787	0.132
Occupation	1.240	0.709	0.224	2.919	0.013*
BMI	1.189	0.580	0.164	2.132	0.027*
Time gastric sleeve surgery done	0.108	0.088	0.124	1.900	0.189
Knowledge	1.284	0.837	0.921	3.422	< 0.001**
Practice	2.470	1.357	1.035	3.137	0.015*

*R* = 0.651

Model ANOVA: F = 7.217, *p*-value < 0.001

Predictors: (Constant), Age (in years), Sex, Educational level, Marital status, Occupation, BMI and time of gastric sleeve surgery, knowledge and practice

Dependent Variable: Total QoL score. * = *p*<0.05, statistically significant. ** = *p*< 0.001, highly significant.

## Discussion

The present cross-sectional study investigated the knowledge, postoperative practices, and quality of life (QoL) of Egyptian patients three to twelve months following sleeve gastrectomy. The results showed that the study population had a mean age of 41.3 years, with females representing the majority of participants. This is consistent with regional and local studies, which indicate that women in this age range are more likely to seek bariatric surgery [13, 14]. These findings suggest that women pursue surgical weight loss interventions due to body image pressures and obesity related comorbidities.

Most participants had a high preoperative weight and a history of chronic illnesses, reflecting the significant burden of obesity related health problems. Previous studies in Egypt and internationally have reported similar findings, highlighting the importance of addressing comorbidities when planning surgical interventions [15, 16, 17]. A considerable proportion had previously attempted weight loss strategies, with motivations for surgery often related to appearance, co-morbidities, or failure of conservative measures, indicating that sleeve gastrectomy is frequently pursued when non-surgical approaches are insufficient [18, 19, 20].

With regards to the postoperative complications were generally low. Immediate complications such as bleeding or infection were rare, and long term issues including constipation, malnutrition, or micronutrient deficiencies affected only a minority of patients. These results align with prior studies reporting relatively low complication rates following sleeve gastrectomy, particularly in settings with structured postoperative care and nutritional follow-up [21, 22, 23, 24].

Regarding knowledge, more than half of the participants demonstrated satisfactory understanding of obesity and sleeve gastrectomy, although some variability exists across studies, likely due to differences in preoperative education programs, health literacy levels, and the quality of counseling [25–26]. From the Health Belief Model perspective, perceived benefits and self-efficacy are critical factors influencing patient adherence. Therefore, structured educational interventions delivered preoperatively and continued postoperatively may facilitate the translation of knowledge into sustained behavioral change.

With regard to postoperative practices, the majority of participants showed satisfactory adherence to dietary, physical activity, and follow up recommendations, consistent with previous studies [27, 28]. According to the Transtheoretical Model and the Health Action Process Approach, maintaining behavior change requires self-regulation and continuous reinforcement. In the Egyptian context, limited access to dietitians and formal postoperative support systems may challenge patients in reaching the maintenance phase. Furthermore, sustained personal motivation and realistic expectations for weight loss may contributing to long term postoperative success. Individuals who view surgery as a supportive tool rather than a permanent solution are more likely to maintain behavioral changes effectively.

Quality of life improved for the majority of participants across physical, psychological, social, and functional domains. These improvements are supported by regional studies, with patients reporting enhanced daily functioning, psychological well-being, and social relationships following surgery [29, 30, 31, 32]. Differences observed between QoL domains in our study may also reflect traditional cultural factors, such as strong family support and collective coping mechanisms, which can enhance psychosocial recovery. Thus, the impact of sleeve gastrectomy on quality of life is influenced not only by the surgery itself but also by the individual’s ability to adapt to lifestyle modifications. However, it should be noted that the follow up period was relatively short (3–12 months), which may limit the assessment of long-term postoperative outcomes. Future studies with longer follow-up are warranted to confirm the sustainability of these results.

There was a statistically significant positive correlation between reported postoperative practices and total quality of life (*r* = 0.441, *p* < 0.001), consistent with previous findings demonstrating that adherence to healthy behaviors is associated with improved well-being after sleeve gastrectomy [33]. This association suggests that sustained adherence to dietary, physical activity, and follow-up practices plays a meaningful role in enhancing postoperative QoL.

From a behavior change perspective, engagement in recommended practices may strengthen self-efficacy and perceived control, thereby supporting both physical and psychosocial recovery. In the Egyptian context, cultural and social resources such as family support, religious coping, and a positive health outlook may further facilitate postoperative adjustment. Nevertheless, individual variation in outcomes highlights the importance of personalized follow-up care, including nutritional counseling, psychological support, and continuous patient education. Overall, the findings emphasize that optimizing long-term quality of life after sleeve gastrectomy requires not only surgical success but also sustained behavioral adherence supported by a multidisciplinary care approach.

## Conclusion

Based on the findings of the present study, most patients demonstrated satisfactory knowledge and acceptable adherence to recommended postoperative practices 3–12 months after sleeve gastrectomy, with a low rate of early complications. Better postoperative practice was strongly associated with improved quality of life, and sociodemographic and clinical factors including sex, occupation, BMI, and baseline knowledge significantly predicted quality of life. These findings highlight the role of structured patient education, behavioral adherence, and multidisciplinary long-term follow-up in optimizing patient centered outcomes after sleeve gastrectomy.

### Implications

Regarding the clinical implications, the health care professionals should strengthen postoperative education by emphasizing the necessity of lifelong adherence to dietary guidelines, vitamin and mineral supplementation, and regular clinical follow-up. Continuous nutritional monitoring, particularly of micronutrients such as iron, calcium, and vitamins B12 and D, should be integrated into routine postoperative care to prevent long-term deficiencies and complications. In addition, for educational implications, structured educational materials and counselling sessions should be developed to raise patients’ awareness of the role of lifestyle modification in achieving and maintaining surgical outcomes. Public health campaigns are also recommended to dispel misconceptions that sleeve gastrectomy allows unrestricted eating, highlighting instead that its success depends on patient commitment and behavior change. Finally, with regard to the research implications, Future studies with larger, multicenter samples and longitudinal follow-up are needed to explore determinants of postoperative adherence and quality of life over time.

### Limitations

This study has several limitations. First, it was conducted in a single outpatient clinic, which may limit the generalizability of the findings to other settings or the broader population of post sleeve gastrectomy patients in Egypt. Second, the follow-up period of 3–12 months may not capture long-term postoperative outcomes. Finally, the sample was purposively selected from the accessible population, which may introduce some selection bias despite efforts to ensure representativeness.

## Data Availability

The datasets generated and/or analyzed during the present study are available from the corresponding author upon reasonable request.

## Abbreviations

QOL: Quality of life
BMI: Body Mass Index
WHOQOL: World Health Organization Quality of Life
SPSS: Statistical package for social sciences
